# Comparison of Efficacy of Intensive versus Mild Pitavastatin Therapy on Lipid and Inflammation Biomarkers in Hypertensive Patients with Dyslipidemia

**DOI:** 10.1371/journal.pone.0089057

**Published:** 2014-02-19

**Authors:** Tomohiro Yamasaki, Yoshio Iwashima, Subrina Jesmin, Yuko Ohta, Hiroshi Kusunoki, Shin-ichiro Hayashi, Takeshi Horio, Yuhei Kawano

**Affiliations:** 1 Division of Cardiovascular Medicine, National Cerebral and Cardiovascular Center, Suita City, Osaka, Japan; 2 Division of Hypertension and Nephrology, National Cerebral and Cardiovascular Center, Suita City, Osaka, Japan; 3 Faculty of Medicine, University of Tsukuba, Tsukuba City, Ibaragi, Japan; 4 Department of General Internal Medicine 3, Kawasaki Medical School, Okayama City, Okayama, Japan; Osaka University Graduate School of Medicine, Japan

## Abstract

**Objective:**

Intensive as compared to mild statin therapy has been proven to be superior in improving cardiovascular outcome, whereas the effects of intensive statin therapy on inflammation and lipoprotein biomarkers are not well defined.

**Methods:**

This study assigned essential hypertensive patients with dyslipidemia to 6 months administration of mild (1 mg/day, n = 34) or intensive pitavastatin therapy (4 mg/day, n = 29), and various lipid and inflammation biomarkers were measured at baseline, and 3 and 6 months after the start of treatment.

**Results:**

Both pitavastatin doses were well tolerated, and there were no serious treatment-related adverse events. After 6 months, significant improvements in total cholesterol, triglycerides, low-density lipoprotein (LDL-) cholesterol, LDL/high-density lipoprotein cholesterol (LDL/HDL), apolipoproteins B, C-II, and E, apolipoprotein-B/apolipoprotein-A-I (Apo B/Apo A-I), and malondialdehyde (MDA-) LDL were observed in both groups. Compared with the mild pitavastatin group, the intensive pitavastatin therapy showed significantly greater decreases in C reactive protein (F = 3.76, p<0.05), total cholesterol (F = 10.65), LDL-cholesterol (F = 23.37), LDL/HDL (F = 12.34), apolipoproteins B (F = 19.07) and E (F = 6.49), Apo B/Apo A-I (F = 13.26), and MDA-LDL (F = 5.76) (p<0.01, respectively).

**Conclusion:**

Intensive pitavastatin therapy may have a more favorable effect not only in decreasing LDL-cholesterol but also in pleiotropic benefits in terms of improvement of apolipoproteins, inflammation, or oxidation.

## Introduction

Hypertension and dyslipidemia, characterized by elevated triglycerides and low density lipoprotein (LDL-) cholesterol with low high density lipoprotein (HDL-) cholesterol, frequently co-exist in the same individual [1], [2], [3]. Simultaneous treatment of two or more risk factors should provide additive benefits in preventing atherosclerotic vascular events, as the results of previous studies have shown that LDL-cholesterol lowering provides a substantial benefit in cardiovascular event reduction even in the presence of good blood pressure control [4], [5]. Pitavastatin is an HMG-CoA reductase inhibitor (statin) that has robust LDL-cholesterol-lowering efficacy at lower doses, and was shown to be noninferior to other statins in terms of improvement of lipid profile [6], [7]. In addition to its effects on the lipid profile, pitavastatin has a number of pleiotropic benefits that reduce residual cardiovascular risk [8], [9], and its beneficial effect in the regression of coronary atherosclerosis in patients with acute coronary syndrome has been reported [10], [11]. Intensive as compared to mild statin therapy has been proven to be superior in improving cardiovascular outcome in clinical trials [12], whereas the relative benefits of such an intensive approach on inflammation, apolipoproteins, and oxidized lipoproteins have not been clarified.

Accordingly, to validate the benefit of intensive lipid-lowering therapy with statins, the present study compared the effects of two different dosages of pitavastatin on inflammation and lipid profile parameters including apolipoproteins and oxidized lipoproteins in hypertensive patients with dyslipidemia.

## Methods

### Study Subjects

This retrospective study enrolled 63 essential hypertensive patients with dyslipidemia with LDL-cholesterol level higher than the National Cholesterol Education Program Adult Treatment Panel III recommendations (<100 mg/dl for moderately high/high-risk subjects without atherosclerotic vascular disease, <70 mg/dl for high-risk subjects with atherosclerotic vascular disease) [13]. Exclusion criteria included age <20 years, treatment for dyslipidemia within the preceding 3 months, current treatment with progesterone or other hormone therapy within the previous 3 months, familial hypercholesterolemia, acute coronary syndrome, congestive heart failure (New York Heart Association class II or greater), liver dysfunction, chronic kidney disease requiring regular hemodialysis, endocrine disease, secondary hypertension, and administration of agents affecting lipid metabolism.

The study protocol was approved by the Institutional Review Board of the National Cerebral and Cardiovascular Center (M25-81). All of the subjects enrolled in this study were Japanese, and all gave written informed consent to participate in this study.

### Baseline Clinical Characteristics

Hypertension was defined as systolic blood pressure (BP) ≥140 mm Hg or diastolic BP≥90 mm Hg on repeated measurements, or receiving antihypertensive treatment. Diabetes mellitus was defined according to the American Diabetes Association criteria [14]. Smoking status was determined by interview, and defined as current, past or never smoker. Previous cardiovascular disease was defined as a history of myocardial infarction, congestive heart failure, or stroke.

### Study Design and Laboratory Measurements

Patients were assigned to two treatment groups with mild pitavastatin therapy (1 mg/day) or intensive pitavastatin therapy (4 mg/day), and were asked to maintain their habitual diet and lifestyle throughout the study. No patient had a change in medication throughout the study period. After fasting overnight, BP was measured with an appropriate arm cuff and a mercury column sphygmomanometer on the left arm after a resting period of at least 10 min in a sitting position. After BP measurement, venous blood and urine sampling from all subjects was performed. Height and body weight were measured, and body mass index was calculated. Data collection was performed at baseline, and at 3 and 6 months after the start of pitavastatin treatment.

Fasting plasma glucose, hemoglobin A1c, serum total cholesterol, triglycerides, apolipoproteins A-I, A-II, B, C-II, C-III, and E, and lipoprotein(a) [Lp(a)] were determined by standard methods, and HDL- and LDL-cholesterol were measured by homogeneous methods (Sekisui Medical Co., Tokyo, Japan). Malondialdehyde (MDA)-LDL was measured by an enzyme-linked immunosorbent assay method (Sekisui Medical Co.) with mouse monoclonal antibody ML25 against MDA residues [15]. The ratios of LDL/HDL and Apo B/Apo A-I were determined by dividing LDL-cholesterol data by HDL-cholesterol, and by dividing apolipoprotein B by apolipoprotein A-I, respectively. The following parameters were also measured: aspartate aminotransferase, alanine aminotransferase, creatine kinase, high-sensitive C-reactive protein, and creatinine. Estimated glomerular filtration rate (eGFR) was calculated using the Japanese coefficient-modified Chronic Kidney Disease Epidemiology Collaboration equation in milliliters per minute per 1.73 meters squared [16], [17]. Urinary albumin excretion was evaluated in each patient by measuring the albumin-to-creatinine ratio (ACR) in first morning samples. Urine albumin concentration was measured by an immunoturbidimetric method. Urine collection was repeated if the patient was menstruating, because this makes albumin measurement unreliable.

The primary outcome was the serial changes from baseline to 3 and 6 months in clinical variables including lipid parameters and biomarkers of inflammation and oxidative stress, and comparisons of serial changes in variables between pitavastatin groups were performed. The secondary outcome was the percentage of patients attaining target lipid levels, defined as LDL-cholesterol <100 mg/dl and LDL-cholesterol <70 mg/dl.

### Statistical Analyses

Data are presented as mean ± standard deviation (SD) for continuous variables, and as actual number for categorical variables unless otherwise specified. First, the significance of any differences between the two groups was evaluated using χ^2^ test for dichotomous variables, and unpaired *t*-test for continuous variables, as appropriate. Because of the right skew of C reactive protein and ACR distributions, levels of these variables were log-transformed to examine the significance of any difference between groups. Second, the significance of differences in parameters before and after administration was evaluated using paired t-test. Third, to determine the significance of the difference in the serial changes in variables by statin administration between groups, repeated measured ANOVA was used. The correlation between baseline variables and their changes by statin administration was assessed by linear regression analysis, and the significance of the difference between two correlation coefficients was assessed using Fisher r-to-z transformation. Finally, comparison of the proportion of subjects achieving the target LDL-cholesterol level between groups was performed by χ^2^ test. All p values were two-sided, and those <0.05 were considered statistically significant. All calculations were performed using a standard statistical package (JMP 8.0; SAS Institute, Cary, NC).

## Results

### Baseline Characteristics and Changes in Lipid Parameters

Baseline clinical characteristics and biochemical parameters of the study subjects are shown in **Tables 1** **and** **2**. Of the 63 participants, 34 were assigned to mild pitavastatin therapy (1 mg/day) and 29 to intensive therapy (4 mg/day). There were no significant differences in baseline characteristics except for lipid parameters between groups. Baseline total cholesterol, LDL-cholesterol (p<0.01, respectively), and apolipoprotein B (p<0.05) were significantly higher in the pitavastatin 4 mg/day group than in the pitavastatin 1 mg/day group (**Table 2**). Both pitavastatin doses were well tolerated without adverse effects, and none of the serious adverse events was considered to be related to pitavastatin.

**Table 1:** Baseline characteristics of study participants.

	Pitavastatin		
	1 mg/day, n = 34	4 mg/day, n = 29	p value
Age, years	68.5±9.9	65.7±12.0	0.32
Male/Female, n	15/19	14/15	0.74
Previous cardiovascular disease, n	18	11	0.23
Myocardial infarction	9	7	0.64
Congestive heart failure	0	2	0.07
Stroke	9	4	0.21
Current smoking, n	3	3	0.84
Diabetes, n	15	11	0.62
Body mass index, kg/m^2^	24.8±3.4	24.3±3.2	0.57
Systolic blood pressure, mmHg	130±14	131±12	0.78
Diastolic blood pressure, mmHg	78±10	74±11	0.12
Heart rate, bpm	73±12	71±12	0.47
Antihypertensive medication			
Calcium channel blocker, n	26	19	0.34
ACEI or ARB, n	32	23	0.07
Beta blocker, n	7	9	0.34
Alpha blocker, n	3	1	0.37
Diuretic, n	11	10	0.86

Values are mean ± SD for continuous variables.

ACEI, angiotensin-converting enzyme inhibitor; ARB, angiotensin II receptor blocker; bpm, beats per minute.

**Table 2:** Lipid, apolipoproteins, and biochemical parameters at baseline and after 3 and 6 months of pitavastatin therapy.

	Pitavastatin 1 mg/day			Pitavastatin 4 mg/day				
	Baseline	3 months	6 months	Baseline	3 months	6 months	F	p‡
Body weight, kg	63.0±12.1	0.1±1.6	0.4±2.7	63.1±12.1	0.1±1.3	0.7±3.0	0.18	0.96
Systolic blood pressure, mmHg	130±14	0.6±12.4	−1.0±13.7	131±12	−3.7±23.7	−6.0±15.3*	0.79	0.46
Diastolic blood pressure, mmHg	78±10	−3.5±8.7*	−4.2±11.2*	74±11	−0.3±7.6	−3.7±6.8^†^	1.33	0.27
Heart rate, bpm	73±12	−2.3±10.9	−0.7±12.0	71±12	−1.3±10.6	−2.4±9.9	0.46	0.63
Aspartate aminotransferase, IU/L	23.9±7.8	2.9±4.2^†^	3.2±6.1^†^	26.1±7.8	2.7±7.2	−1.1±13.4	1.91	0.15
Alanine aminotransferase, IU/L	21.7±15.1	3.50±5.4^†^	4.4±10.8*	26.7±15.4	3.9±10.7	−0.8±12.5	2.76	0.07
Creatine kinase, IU/L	100±51	17±39*	27±96	114±59	37±237	1±53	0.85	0.43
eGFR, ml/min/1.73 m^2^	69.8±17.4	−1.8±4.8*	−1.6±5.3	72.8±17.3	−2.9±5.9*	−1.8±4.5*	0.45	0.64
ACR, mg/g creatinine, median (IQR)	13.5	−0.2	0.7	7.3	−1.2	−1.7	0.26	0.77
	(5.8, 44.0)	(−6.9, 3.6)	(−7.8, 7.0)	(4.6, 61.9)	(−14.2, 3.3)	(−48.7, 3.6)		
Hemoglobin A1c, %	6.0±0.6	0.2±0.4	0.2±0.3	6.1±1.1	0.0±0.5	0.2±0.4	0.41	0.67
C reactive protein, mg/dL, median (IQR)	0.09	−0.01	0.02	0.09	−0.04	−0.03	3.76	<0.05
	(0.04, 0.14)	(−0.05, 0.04)	(−0.03, 0.10)	(0.07, 0.31)	(−0.09, 0.00)*	(−0.12, 0.00)*		
Total cholesterol, mg/dL	225±6	−42±34^†^	−46±31^†^	252±7	−69±43^†^	−82±37^†^	10.65	<0.01
Triglycerides, mg/dL	147±54	−11±48	−23±43^†^	138±45	−11±59	−26±33^†^	0.04	0.96
HDL-cholesterol, mg/dL	53±12	1.9±6.8	2.7±8.7	57±17	5.0±18.3	2.9±8.7	0.79	0.46
LDL-cholesterol, mg/dL	143±31	−39±29^†^	−38±28^†^	169±34	−72±26^†^	−80±30^†^	23.37	<0.01
LDL/HDL	2.83±0.83	−0.87±0.60^†^	−0.87±0.61^†^	3.23±1.08	−1.51±0.88^†^	−1.61±0.84^†^	12.34	<0.01
Apolipoprotein A-I, mg/dL	140±18	5.2±11.6*	7.2±15.3*	145±33	12.3±26.1*	8.6±15.9^†^	1.48	0.23
Apolipoprotein A-II, mg/dL	30±5	1.04±2.56*	0.97±3.13	30±7	1.57±4.95	1.04±3.81	0.21	0.81
Apolipoprotein B, mg/dL	110±18	−27.6±15.6^†^	−29.3±15.7^†^	122±22	−45.2±16.7^†^	−51.8±18.9^†^	19.07	<0.01
Apo B/Apo A-I	0.80±0.16	−0.23±0.12^†^	−0.24±0.12^†^	0.88±0.25	−0.37±0.19^†^	−0.41±0.20^†^	13.26	<0.01
Apolipoprtein C-II, mg/dL	5.48±1.54	−0.54±1.13^†^	−0.71±1.31^†^	5.67±1.62	−1.08±1.31^†^	−1.41±1.17^†^	2.99	0.054
Apolipoprotein C-III, mg/dL	10.8±2.9	−0.03±2.96	−0.65±2.36	11.0±3.5	−0.21±2.99	−1.00±1.78^†^	0.17	0.85
Apolipoprotein E, mg/dL	4.14±0.77	−0.50±0.67^†^	−0.64±0.66^†^	4.35±0.79	−0.90±0.55^†^	−1.14±0.57^†^	6.49	<0.01
Lp (a), mg/dL	20.63±16.93	−0.95±4.94	−0.07±6.73	29.9±29.18	0.39±9.71	1.04±7.94	0.29	0.75
MDA-LDL, U/L	161.3±50.0	−35.4±49.3^†^	−35.8±50.0^†^	180.7±50.3	−63.3±48.6^†^	−72.1±45.7^†^	5.76	<0.01

Values are mean±SD or median (IQR). IQR is 25th to 75th percentile.

ACR, albumin-to-creatinine ratio; bpm, beats per minute; eGFR, estimated glomerular filtration rate; HDL-cholesterol, high-density lipoprotein cholesterol; IQR, interquartile range; LDL-cholesterol, low-density lipoprotein cholesterol; Lp (a), lipoprotein(a); MDA-LDL, malondialdehyde-LDL.

*p<0.05 and ^†^p<0.01 versus baseline.

‡ p values of repeated measures ANOVA.

**Figure 1:**
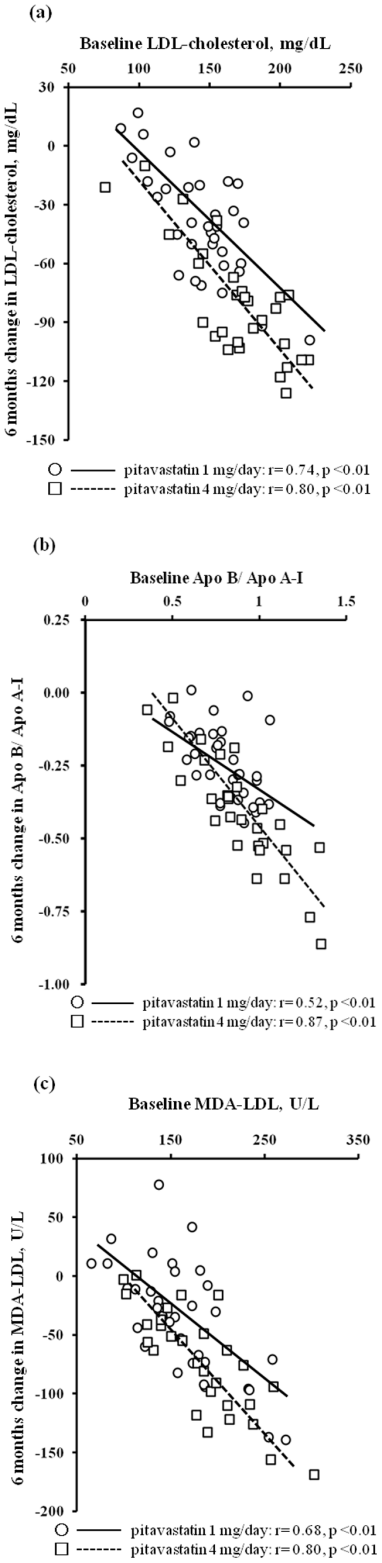
Correlation between baseline LDL-cholesterol (a), Apo A-I/Apo B (b), and MDA-LDL (c), and their changes after 6 months of pitavastatin treatment. Open circles indicate patients with pitavastatin 1/day; open squares indicate patients with pitavastatin 4 mg/day.

Serial changes in biochemical parameters after pitavastatin treatment are shown in **Table 2**. Both doses significantly decreased diastolic BP, total cholesterol, triglycerides, LDL-cholesterol, LDL/HDL, apolipoproteins B, C-II, and E, Apo B/Apo A-I, and MDA-LDL, and significantly increased apolipoprotein A-I. At 6 months after the start of treatment, systolic BP, C reactive protein, apolipoprotein C-III, and eGFR were significantly decreased in the pitavastatin 4 mg/day group. When compared with pitavastatin 1 mg/day, pitavastatin 4 mg/day showed significantly greater decreases in C reactive protein, total cholesterol, LDL-cholesterol, LDL/HDL, apolipoproteins B and E, Apo B/Apo A-I, and MDA-LDL over the first 3 and 6 months. Serial changes in other variables were not significantly different between the two groups. The correlations between baseline LDL-cholesterol, Apo B/Apo A-I, and MDA-LDL, and their changes after 6 months of pitavastatin treatments are shown in **Figure 1**. When compared with pitavastatin 1 mg/day, pitavastatin 4 mg/day showed a significantly higher correlation coefficient for Apo B/Apo A-I (p<0.01), but not for LDL-cholesterol (p = 0.30) and MDA-LDL (p = 0.15).

We next repeated our analysis in the 29 patients with previous cardiovascular disease. Even in this subgroup, at 6 months after the start of treatment, both doses significantly decreased LDL-cholesterol (pitavastatin 1 mg/day, 125±28 to 101±25 versus 4 mg/day, 152±41 to 83±22 mg/dL), Apo B/Apo A-I (pitavastatin 1 mg/day, 0.74±0.17 to 0.56±0.18 versus 4 mg/day, 0.81±0.25 to 0.45±0.09), and MDA-LDL (pitavastatin 1 mg/day, 160±52 to 120±35 versus 4 mg/day, 172±63 to 85±19 U/L) (p<0.01, respectively). When compared with pitavastatin 1 mg/day, pitavastatin 4 mg/day showed significantly greater decreases in LDL-cholesterol (F = 11.27, p<0.01), Apo B/Apo A-I (F = 4.63, p = 0.01), and MDA-LDL (F = 3.21, p = 0.046) over the first 3 and 6 months.

### LDL-cholesterol Attainment

At baseline, the proportion of patients achieving LDL-cholesterol <100 mg/dL was not significantly different between the groups (pitavastatin 1 mg/day, 8.8% versus 4 mg/day, 3.5%), and none of the patients achieved LDL-cholesterol <70 mg/dL. At the end of 6 months of pitavastatin treatment, the proportion of patients achieving LDL-cholesterol <100 mg/dL was significantly higher with pitavastatin 4 mg/day than with 1 mg/day (69.0% versus 44.4%, p<0.05). The proportion of patients achieving LDL-cholesterol <70 mg/dL was also significantly higher with pitavastatin 4 mg/day than with 1 mg/day (20.7% versus 2.9%, p<0.05).

## Discussion

The present study compared the effects of two different dosages of pitavastatin on a variety of established and emerging lipid profile parameters. Although significant improvements in diastolic BP, total cholesterol, triglycerides, LDL-cholesterol, LDL/HDL, apolipoproteins A-I, B, C-II, and E, Apo B/Apo A-I, and MDA-LDL were observed in both groups, compared to mild pitavastatin therapy, intensive pitavastatin therapy showed a greater reduction in total cholesterol, LDL-cholesterol, C reactive protein, LDL/HDL, apolipoproteins B and E, Apo B/Apo A-I, and MDA-LDL.

Our results were partially in accordance with previous findings that pitavastatin treatment resulted in an improved lipid profile in terms of providing improvement from baseline in total cholesterol, triglycerides, and LDL-cholesterol, and the lipid-modifying effects in this study were also roughly in agreement with previous findings [7], [9]. Pitavastatin has high affinity to the hydrophobic regions of HMG-CoA reductase [7], and inhibits HMG-CoA reductase [18] as well as cholesterol synthesis in cultured hepatic cells in a dose-dependent fashion [19]. The therapeutic efficacy of pitavastatin has been evaluated in patients with primary hypercholesterolemia, mixed dyslipidemia [20], the elderly [7], and type 2 diabetes [21]. This study extended these favorable lipid-modifying effects of pitavastatin to hypertensive patients with dyslipidemia, and showed dose-dependent improvement in the lipid profile.

Consistent with prior findings, this study showed that pitavastatin treatment was associated with significant improvement in apolipoprotein parameters: apolipoproteins A-I, B [20], C-II, and E, and Apo B/Apo A-I. Apo B/Apo A-I has been shown in large prospective studies to be an indicator of cardiovascular risk. Decreased secretion of apolipoprotein B from hepatoma cells has been reported in the presence of pitavastatin [22], and pitavastatin was more potent than simvastatin or atorvastatin in inducing apolipoprotein A-I secretion [23]. Apolipoprotein A-I is the major protein of HDL-cholesterol, and apolipoprotein B is the major apolipoprotein of the atherogenic lipoprotein families very low density lipoprotein lipoprotein (VLDL), intermediate-density lipoprotein (IDL), and LDL-cholesterol. The concentration of apolipoprotein B is a good estimate of the number of these particles in plasma, and measurement of apolipoprotein B represents the total burden of particles considered most atherogenic [24], [25]. Apolipoprotein B has been found to be a better predictor of cardiovascular risk than is LDL-cholesterol in several epidemiological studies and in post hoc analyses of clinical trials [26], [27], particularly the on-treatment LDL-cholesterol level [27], [28], suggesting a more effective way to assess residual cardiovascular risk and to determine the need for medication adjustment in patients receiving LDL-lowering therapy.

In this study, intensive pitavastatin therapy also showed more favorable effects in reducing the inflammatory or oxidative response, assessed by C reactive protein and MDA-LDL levels [29], which may play a pivotal role in all stages of atherosclerosis. Therefore, our results suggest that, compared with mild pitavastatin therapy, intensive pitavastatin therapy has more beneficial pleiotropic effects in terms of improvement in apolipoproteins, inflammation, or oxidation.

In this study, no severe treatment-related adverse events were observed. With regard to the tolerability of pitavastatin, a two year prospective study of over 20,000 patients treated with pitavastatin revealed no unexpected negative side effects of treatment [30]. Although all statins, to a greater or lesser degree, have the potential to cause adverse events if administered in sufficiently high doses, or in combination with other medications that alter their pharmacokinetics, pitavastatin is minimally metabolized by cytochrome P450 (CYP) isoenzymes [8] and has a low possibility of interaction with those drugs metabolized by, inhibiting or inducing CYP enzymes, thereby having a low propensity for drug-drug interactions. This low propensity for drug-drug interaction is important especially for patients requiring polypharmacy because combination therapy with statins and other agents is likely to become increasingly common, both to achieve lipid targets and to treat comorbid conditions.

There are several limitations of this study. This study was not randomized and not blinded; however, the outcome was objective and was assessed in a blinded manner. A cross-over trial may be more appropriate to prove our hypothesis. In this study, the two treatment groups were not well matched in terms of baseline plasma lipid levels including LDL-cholesterol, and the study was of modest size and conducted for a relatively short time. Most trials that have investigated the favorable effects of intensive statin therapy were performed in high-risk patients with a substantially high cholesterol level or with evidence of existing cardiovascular disease. Further studies are needed to validate whether intensive statin therapy also leads to a greater reduction in cardiovascular events even in patients with essential hypertension.

In conclusion, intensive pitavastatin therapy was more effective than mild therapy in improving atherogenic risk factor profiles, indicating that it not only has stronger LDL-cholesterol-lowering effects but also has stronger pleiotropic effects in improvement of apolipoproteins, inflammation, or oxidation than does mild therapy. The similar safety profile suggests that pitavastatin 4 mg/day may be preferable in high-risk patients in whom the target has not been achieved with other statin therapy, and in those requiring polypharmacy, such as patients with hypertension.
